# Implicit Messages Regarding Unhealthy Foodstuffs in Chinese Television Advertisements: Increasing the Risk of Obesity

**DOI:** 10.3390/ijerph15010070

**Published:** 2018-01-04

**Authors:** Angela Chang, Peter J. Schulz, Tony Schirato, Brian J. Hall

**Affiliations:** 1 Department of Communication, University of Macau, E21, Avenida da Universidade, Taipa, Macau, China; tonyschirato@umac.mo; 2 Institute of Communication and Health, Lugano University, Switzerland, Ex Laboratorio, Office 010 (Level 0), Via Buffi 13, 6904 Lugano, Switzerland; peter.schulz@usi.ch; 3 Department of Psychology, University of Macau, E21, Avenida da Universidade, Taipa, Macau, China; brianhall@umac.mo; 4 Department of Health, Behavior and Society, Johns Hopkins Bloomberg School of Public Health, Baltimore, MD 21205, USA

**Keywords:** big foods, branding strategy, advertising appeals, advertising tactics, obesity, food marketing, global brands

## Abstract

Previous studies indicated that television (TV) advertising is associated with higher rates of obesity. The rate of obesity and overweight continues to rise in mainland China, bringing into question whether TV advertising to young audiences might be partly to blame. This study investigated messaging delivered through TV advertisements regarding healthy and unhealthy foodstuffs. A total of 42 major food brands and 480 advertisements were analysed for content in this study. The results showed that the majority of TV spots advertised products with poor nutritional content and had a potential to mislead audiences concerning products’ actual nutritional value. The tactics of repetition and appeals of premium offerings on food brands have a potential to influence the purchase intentions. Additional qualitative observation involving the social bond, social context and cultural factors pertaining to mood alterations were highlighted. The discussion addressed product attributes reflected by culture and the implicit messages of marketing claims may increase the risk of obesity. Thus, public health policymakers and researchers were encouraged to act urgently to evaluate the obesity risks of unhealthy food advertised in the media and to support healthy foods.

## 1. Introduction

The increasing rate of obesity in mainland China has become a major concern. China’s first official nutrition and health survey reported that between 1992 and 2002, approximately 4.8 million children living in China’s cities were classified as obese [1,2,3]. The prevalence of overweight and obese children aged 7–18 years increased to 30.4 million in 2010. From 1985 to 2010, there was a significant and continual increase in the prevalence of obesity in children, which is now consistent with the rates in developed countries [4,5,6]. A more recent report revealed that childhood obesity in China has drastically increased due to frequently consuming sugared drinks [7]. These data suggest that obesity rates are likely to increase, especially among Chinese children and youth. 

Mainland China has recently experienced a surge in childhood obesity. This is partially due to the developing market economy, which is transforming the food environment and food consumption patterns. This is evident in the major dietary changes in China over the past 20 years, which involve moving from a diet primarily based on cereal grains and vegetables to one in which the consumption of products that are high in sugar, oil, or other fats has increased [8,9,10,11,12]. The food industry is clearly a major factor in the increase in obesity, as food manufacturers, processors, and retailers have considerable power to increase market access and influence eating habits through the products they choose to sell, retail pricing, and the labelling and promotion of particular goods [13,14].

The quality and types of food potentially promoted in media by the food industry and perceived by consumers undermining food preference and consumption. Therefore, this study focused on the variety of food products advertised in the Chinese market by investigating advertising tactics, appeals, presentation manner, primary themes, and health-related claims for a range of food categories. The aim of the study was to uncover the implicit messaging featured in TV advertisements (ads) and fill the gap in knowledge regarding the range of unhealthy food products in TV advertising and branding. Certain branded products might intend to promote local adaptation of divergent social aspirations and cultural values. 

### 1.1. Food Advertising in China

An annual global report on media trends shows that China has overtaken Japan as the second largest advertising market since 2012 in Asia [15]. Total advertising expenditure in China reached USD50.17 billion in 2014 and is expected to grow to USD66.99 billion by 2018. Specifically, TV ad spending in China was close to USD46.5 billion in 2015 [16]. In addition, foreign food companies are establishing a considerable presence in China due to its large consumer market (1.3 billion people) and rapid economic growth (9% per year) [17]. The food industry is one of the major players in the field of advertising. In particular, the multinational food and beverage corporations, with their enormous and concentrated marketing power for ultra-processed packaged food and beverages, are comprised of ‘big food’ companies [18]. For example, more than half of global soft drinks are produced by large companies (e.g., Coca-Cola, Pepsi, Atlanta, GA, USA), thus criticism of these “big food” companies has addressed behaviors such as exploiting schools for commercial gain and producing or promoting large portions of sweetened beverages among children, as these increase the risk of obesity [19,20,21]. 

Previous studies show that TV food ad viewing has been linked to obesity through several complicated factors, such as an individual’s dietary choices, sedentary lifestyle, eating habits, and the way in which promotional techniques are understood to employ affect and emotion [22,23,24]. Parvanta et al. [25] examined the association of TV watching and paying attention to TV ads with buying and requesting snacks seen in ads, as well as eating snacks while watching TV, among youth in China. They concluded that paying attention to TV ads for snack foods may be one of the significant factors affecting the increase in obesity among children in China. Another study for children and youth aged 6–18 years by Cui, Hardy, Dibley and Bauman [26] confirmed that TV viewing time and sedentary behavior have significantly increased from 1997 to 2006 in China, which were similar to those youth in America or France. Further, their surveys concluded a strong positive association between parent-child co-viewing TV and the child’s screen time by suggesting parents’ role modelling and interventions for both child and parental TV viewing.

Advertising has been considered to be an important socialization agent from which young consumers learn certain values and skills. Studies have evidenced that the different content of TV ads contributed to differences in children’s socialization experiences [27,28,29]. For instance, children exposed to TV ads for junk food were found to be more likely to consume food high in sugar and fat, which contributes to the increase in obesity in China [25] and in South Africa [19]. Moreover, advertising less healthy food to children tended to encourage less critical attitudes towards the advertised food among children [30,31]. 

Research has shown that people who are constantly exposed to TV food ads tend to consume more unhealthy snacks and thus more calories [32,33,34]. The causal linkages in media discourse has been made explicitly or implicitly. Indeed, advertising—especially TV advertising—has been identified as a major contributor in promoting unhealthy eating habits and childhood obesity. The considerable body of research on the effects of TV advertising on childhood obesity has been largely limited to developed Western countries [30,32,33,35,36]. However, little attention has been paid to advertising activities and their influence on obesity, and on childhood obesity in particular, in less developed countries or cultures. 

Several studies have examined the food products represented and promoted on TV, identifying food product types commonly associated with unhealthy diets [37,38]. For example, fast food restaurants, sweetened soft drinks, and sweetened cereals dominated TV food advertising in Switzerland [39]. Powell et al. [36] examined the nutritional content of five food categories seen in TV ads by American children and adolescents in terms of fat, saturated fat, sugar, sodium, and fibre. Their results showed that 97.8% and 89.4% of advertised food products viewed by children and adolescents, respectively, were high in fat, sugar, or sodium. Research has also indicated that calorie-dense foods, such as fast food and sweetened soft drinks, are more easily accessible than fruits and vegetables [26,29,40]. This is attributed to the relatively low price of mass-produced dairy products and processed food due to economy-of-scale production, technological innovations in food processing and storage, and government-subsidized food production. 

### 1.2. Persuasive Messaging Strategies 

Food manufacturers use a range of promotional strategies to sell their products. For example, emotional appeals (e.g., mood alteration, fun) and sensory references (taste/flavour/smell/texture) without communicating pertinent product information were frequently employed [38]. The majority of American TV ads have been found to promote food of low nutritional quality, yet over half contain an implicit health-related message [41,42]. Most ads for nutritionally deficient food (e.g., fast food and sweetened cereals) have been found to contain appeals to healthiness/nutrition [38]. This may mislead perceptions regarding the actual nutritional value of problematic food products. 

Kunkel and Gantz [43] analysed TV ads from three national American networks—one independent broadcaster and two cable channels that target children—defining five categories of advertising appeals. Their findings indicated that fun, happiness, and free gift incentives (“premiums”) appeared frequently. Page and Brewster [35], in turn, listed 20 advertising tactics in TV ads for fast food and pizza restaurants that appeared during children’s programs on American broadcast networks. Their findings indicated that jingles or slogans and pictures of children with food were the most frequently used tactics by which viewers could identify with advertised products. Animations, peers, and animal characters were also used to attract attention [35,38]. 

The tactics and appeals of TV advertising are somewhat different in the Chinese market compared to the market in Western culture [44]. For example, Liu et al. [17] surveyed food ads in mainland China, proposing that appeals for local products usually emphasized their utilitarian functions and durability, whereas foreign products were evaluated according to their symbolic value, such as signifying modernity, status, taste, and technology. Ji and McNeal [27] indicated that Chinese children’s TV ads mainly showed products in use to avoid uncertainty. Their study also revealed that ad products recommended/endorsed by opinion leaders, well-known people, or family elders was one of the most frequently used techniques by following the Chinese tradition of authoritative culture. In comparison, American spots emphasized product texture or quality and portrayed appeals toward fun, happiness, or adventure [45,46,47]. The most frequently used techniques in Western ads for unhealthier food products, “premium offerings” and “promotional characters” were not applied to a similar degree in Chinese culture [27]. This is presumably because the majority of child-targeted food ads seemed to take a branding approach, focusing on creating lifelong customers rather than generating immediate sales [1,46].

Aside from the effects of TV advertising on children, food ads targeting parents have also drawn attention from scholars. Emond et al. [45] compared food ad characteristics and patterns between parents and children in America. Their result showed that nutritionally deficient children’s foods were frequently advertised to parents by employing emotional (e.g., mood alteration, fun) and health-related appeals. Bernhardt et al. [47] concluded that fast food aimed at children emphasized toy premiums and movie tie-ins, brands and logos while in comparison, adult-directed ads emphasized the taste, portion size and price of fast food products. 

In sum, previous findings showed that children in some developed countries such as America and Switzerland were targeted with food advertising messages that urged the consumption of unhealthy foods [25,37,38,39]. For an overview of the broad environmental determinants of diet and the supply of local and global foodstuffs, one hypothesis was proposed:

**Hypothesis** **1** **(H1).** *Unhealthy food products are frequently advertised in China*.

Given the potential impact of unhealthy foodstuffs and their advertising messages on children’s dietary behavior, the most frequently emphasized tactics in food ads included premium offerings [35,43]. Thus, one hypothesis was proposed:

**Hypothesis** **2** **(H2).** *Unhealthy food ads tended to frequently use premium offerings, when comparing healthy foodstuffs or unhealthy foodstuffs in China*.

Furthermore, the majority of nutritionally deficient foods employed sensory appeal (taste/flavor/smell/texture) in food ads [38]. Thus, one hypothesis was proposed:

**Hypothesis** **3** **(H3).** *Unhealthy food ads tended to frequently use sensory appeals, when comparing healthy foodstuffs or unhealthy foodstuffs in China*.

Considering the local adaptation of divergent social aspirations and cultural values, Chinese TV ads tended to show products in use and endorsed by public figures or elders, instead of emphasizing product texture and symbolic values [22,27,46]. Thus, one hypothesis was proposed:

**Hypothesis** **4** **(H4).** *Local brand ads tended to use product demonstration and endorsed products as advertising tactics, when comparing local and global brand origin in China*.

## 2. Materials and Methods

### 2.1. Data Collection

The food brands sampled in this study were obtained from the World Brand Lab. This database lists the world’s 500 most valuable brands, as well as China’s 500 most valuable brands. The brand was recruited on the basis of financial status with revenue, earnings, growth potential, and brands value estimated in currency on the top of brand list [48]. Excluding non-food brands, 42 brands (30 local Chinese and 12 global brands) were identified for collecting TV ads (See Supplementary Materials). 

The sample of TV ads used in the present analysis was obtained from the AD Topic database [49] in 2012. The AD Topic database is a free access website with a collection of over 20,000 local and global TV ads dating from 2007. All the sampled TV ads were broadcast on nationwide channels (e.g., China Central TV) or cable networks (e.g., Hunan Satellite TV). As more TV advertising was making its way to the websites, the sampled TV ads also could be found in the format of web video ads from the advertisers’ official websites. All the sampled TV ads represent commercials from a wide variety of food products and food product categories. The same TV ads appeared to air on the nationwide broadcast networks, cable networks, and websites of advertisers and advertising database. 

Ads were categorized as child-targeted if one of six criteria were met by considering child-directed advertising content [38,45,46,47] and cultural factors [27,50,51]: (1) the ad was placed on a children’s program; (2) children appeared visually in the ad; (3) child only or child-adult in voice-overs; (4) men as models and spokespersons; (5) the ad contained verbal references or appeals to children; or (6) the ad promoted products specifically marketed to children. Duplicate ads from the database were included only once. 

### 2.2. Measures 

All measures used in this study were adopted from established categories in the literature. Each food ad was coded in terms of the following eight variables: origin of brand (global vs. local) [27], product category [27,50], healthiness [30,50], advertiser’s tactics [51], persuasive appeals [38,51], health-related claims [36,50], manner of presentation [35,50], and primary theme [43,45,46,52]. 

Advertised foods were classified as either healthy or unhealthy in accordance with previous studies. Unhealthy food is defined as food that should be limited in a balanced diet or consumed in moderation because of its energy-dense, nutrient-poor characteristics and association with obesity [30,50,53]. In comparison, healthy food is defined as government recommends this food, comprising essential vitamins and minerals, or nutritional levels of saturated fat, sodium, and sugar [30,47,53]. Where necessary, information required to classify advertised products was obtained by checking the website of a particular food company or the product label information on ingredients. Appendix A (Table A1) lists healthy versus unhealthy foods, gives definitions and instruction details regarding the coding categories and the results of inter-coder reliability measures. 

Advertisers’ tactics, persuasive appeals, presentation techniques, and themes could potentially each be coded more than once for a given ad, as an ad might use more than one appeal or tactic. However, brand origin, product category, healthiness, and health-related claims were coded only once, as these were mutually exclusive. Three Chinese graduate students majoring in communications and new media were trained over 10 h to act as independent coders. Each coder was required to code at least 200 ads, provide dichotomous judgments, and indicate uncertainty on the coding sheet. If an ad provided a visual presentation or verbal statement suggesting a health benefit of a nutrient or other substance in the product, it was coded for the presence of a healthy food claim. Coders discussed all disagreements and uncertainties with the first author during the final stage of the analysis. Previous studies have recommended 80% inter-coder agreement as an acceptable minimum [50,54]. The inter-coder reliability for each variable in the present study indicated substantial agreement across coders (with Cohen’s Kappa ranges from 0.81 to 1.0) (*p* < 0.01), indicating high confidence in the reliability of the findings [55]. 

### 2.3. Data Analysis 

TV ad qualities were described and cross-tab analysis was used to compare the frequency difference between healthy and unhealthy foodstuffs as well as global and local Chinese brands. Frequencies and percentages were calculated to summarize the distributions of the food or ad categorical variables. All quantitative analyses were completed with IBM Statistical Package for the Social Sciences, version 22 (IBM Corp., Armonk, NY, USA). Chi-square analysis were used to test hypothesis regarding categorical by categorical associations [27,45,47,50]. 

Roberts and Pettigrew [52] proposed that the existing content analysis literature provides abundant information in terms of quantifying food products and advertising appeals depicted in TV ads. However, little is known about the implicit messages with symbolic values delivered through food ads and the ways this might affect consumers’ perceptions and interpretations of such messages [29]. We followed Roberts and Pettigrew’s example and complemented our quantitative results with more qualitative observations. 

## 3. Results

### 3.1. Unhealthy Food Featured

Only a minority (*n* = 133, 27.7%) of the 480 TV ads analysed in this study advertised healthy food, while most ads were for unhealthy food (*n* = 347, 72.3%). Thus, H1 that proposed that unhealthy food products are frequently advertised in China was supported. Fast food ads comprised over one-third of all ads; this was the most frequently advertised food category, followed by four categories with a similar distribution: fruit or vegetable juice and water (*n* = 59, 12.3%); dairy (*n* = 57, 11.9%); sweetened beverages (*n* = 54, 11.3%); and cookies, desserts, and snacks (*n* = 50, 10.4%). Among 13 ads of rice, grain, cereals, and pasta product, there was only one ad found for the unsweetened cereals. Additionally, there were no ads for meats/fish/poultry, condiments, or cakes/pastries. The ads of sweetened drink featured a variety of 21 local and seven global brands, whereas fast food ads were predominated by three global brands: McDonald’s (Oak Brook, IL, USA), KFC (Louisville, KY, USA), and Pizza Hut (Plano, TX, USA) (Table 1). 

### 3.2. Advertising Tactics and Persuasive Appeals

Table 2 provides a comparison of the advertising tactics used and the persuasive appeals, both categorized in terms of healthy and unhealthy food groups. Advertising for healthy food relied heavily on product demonstration, at 83.5% (*n* = 111). Healthy food ads tended to use three of the advertising tactics less frequently (i.e., peer popularity, premium offerings, and humour) than did those for unhealthy food. Instead, the most significant difference in advertisers’ tactics involved premium offerings, at 83.8% (*n* = 289), and humour employed, at 71.5% (*n* = 248), in spots for unhealthy foodstuffs ($x^{2}$ = 186.023, df = 5, *p* < 0.01). In comparison, unhealthy food tended to use more premium offerings (83.8%, *n* = 289) than healthy food (24.8%, *n* = 33). Thus, H2 that proposed that unhealthy food ads tended to frequently use premium offerings when comparing healthy foodstuffs or unhealthy foodstuffs in China was supported.

The overall persuasive appeals of food ads emphasized the novelty aspects of the products (65.6%, *n* = 315), and their association with strength, speed, or power (60.0%, *n* = 288). Specifically, unhealthy food ads especially focused on novelty aspects (85.9%, *n* = 298), followed by strength, speed, or power (67.4%, *n* = 234). The persuasive appeals of healthy food ads focused on nutritional claims (65.4%, *n* = 87) and goal achievement (51.1%, *n* = 68) ($x^{2}$ = 361.35, df = 5, *p* < 0.01). In comparison, unhealthy food tended to use more sensory appeals (taste/flavor/smell/texture) (58.2%, *n* = 202) than healthy food (44.4%, *n* = 59). The majority of unhealthy food ads tended to frequently use sensory appeals in TV ads, when comparing healthy foodstuffs or unhealthy foodstuffs in China, thus, H3 was supported. 

Table 3 shows the advertising tactics and persuasive appeals by brand reach (local or global). Local brand advertising relied heavily on repetition, at 74.4% (*n* = 148), followed by peer popularity, at 56.8% (*n* = 113), whereas global brand advertising emphasized peer popularity, at 59.1% (*n* = 166), followed by repetition, at 58.0% (*n* = 163), and product demonstration, at 49.8% (*n* = 140) ($x^{2}$ = 81.22, df = 5, *p* < 0.01). In comparison, global brands tended to use more product demonstration (49.8%, *n* = 140) than local brands (35.2%, *n* = 70). Thus, H4 that proposed that local brand ads tended to use product demonstration for one of the advertising tactics when comparing local and global brand origin in China was not supported.

As to appeals, both local and global brand groups emphasized food’s tastiness equally. The global brands placed a strong emphasis on taste, flavour, smell, or texture (55.9%, *n* = 157) and novelty (40.2%, *n* = 113), whereas taste, flavour, smell, or texture (55.3%, *n* = 110) and nutritional value (39.7%, *n* = 79) were emphasized by the local brands ($x^{2}$ = 95.717, df = 5, *p* < 0.01). Thus, H4 that proposed that local brand ads tended to use product endorsement for advertising tactics, when comparing local and global brand origin in China was partially supported.

### 3.3. Manner of Presentation and Primary Themes

Table 4 displays the comparison between the ads for healthy and unhealthy food products in terms of the manner of presentation. Advertising slogans were the most frequently used in approximately 83.5% (*n* = 111) and 86.2% (*n* = 299) of ads for healthy and unhealthy foodstuffs, respectively. Jingles (74.6%, *n* = 259) were the second most frequently used technique employed in unhealthy food ads, whereas celebrity endorsement or mascots (44.4%, *n* = 59) were often adopted for healthy food ads ($x^{2}$ = 70.43, df = 4, *p* < 0.01). 

The primary theme of happiness (66.9%, *n* = 89) was prevalent in ads for healthy foodstuffs whereas the theme of mood alterations (86.7%, *n* = 301) was frequently used for unhealthy food ads. Family togetherness was the second most frequently adopted primary theme (66.2%, *n* = 88) for healthy food, whereas the other three themes were similarly emphasized for unhealthy food products, except the theme of family togetherness ($x^{2}$ = 183.10, df = 4, *p* < 0.01).

### 3.4. Further Observations

Starting with the quantitative results, additional observations on characteristics of food advertising in China seemed to be noteworthy. Despite a wide array of brands available, TV food advertising was dominated by the big food companies’ brands: Coca-Cola, PepsiCo (Purchase, NY, USA), Kellogg’s (Battle Creek, MI, USA), Mars (McLean, VA, USA), Kraft (Chicago, IL, USA), Nestle (Vevey, Switzerland), Master Kang (Kangshifu) (Tianjin, China), Tongyi (Shenzhen, China), Yili (Hohhot, China), and the Wahaha (Hangzhou, China). These brands generated from transnational or China local food and beverage corporations targeted mainland China markets with vigour. In 2013, Wilmar (Kellogg’s) from Singapore triumphed regional sales of USD11.6 billion in China; Coca-Cola, Pepesi, Mondeles (Kraft), and Mars from the US reached USD8.7, 4.5, 4.1, and 2.6 billion, respectively; Nestle from Switzerland reached USD2.9 billion; additionally, McDonald’s and biggest rival Yum China Holdings Incorporation, which operates the KFC and Pizza Hut brands in mainland China reached USD25.9 billion of sales, approximately 50% of world sales [13].

The second observation involved the social bond associated with the advertised foodstuffs, sometimes directly depicted as a situation occurring when the food was consumed. Family togetherness in 31.0% (*n* = 149) of all the ads was presented with the least prevalence of the primary themes measured in this study. Emond and others [45] have proposed the importance of family bonding appeal in parent-directed television ads for children’s packaged foods and beverages. Roberts and Pettigrew [52] have already shown in the Australian context that family meals were the least common type of eating situation portrayed, appearing in only 7% of ads. In our study, the portrayal of family togetherness, including images of shared family meals, occurred far more commonly in promotions of healthier foodstuffs (66.2%, *n* = 88) than the unhealthier foodstuffs (17.6%, *n* = 17.6). Additionally, while being with peers or friends tended to go along with the consumption of snacks, chocolate bars, sweets, and cold beverages as a fun activity in spots propagating unhealthy foodstuffs (83.3%, *n* = 289) than the healthy foodstuffs (42.1%, *n* = 56). 

An additional example pertaining to the social context of branded food ads was the promotion of local adaptation. The local Chinese brands seemed to associate well-being and happiness with togetherness, while the ads for global brands appeared to link happiness and good feelings with solipsism and solitary eating. The most notable image of popularity through snacking was an ad for a local brand, Master Kang instant noodles, in which teenagers were depicted as greatly enjoying the food while sharing the noodles with peers and friends on various occasions. 

The last observation, likely reflecting a cultural factor, pertained to mood alterations as a predominant theme (86.7%, *n* = 301) for unhealthy foodstuffs. Mood alterations seemed to go along with increased surprise, and the respective ads tended to share basic similarities in terms of style and content. The ads promoting food and beverages to children typically featured youth dancing, singing, excitement, fast action, and enjoyment. The mood improvement mainly implied either shared or solitary emotional highs from consumption of the product. 

## 4. Discussion 

### 4.1. Product Attributes Reflected by Culture

The present study showed that the majority of TV ads for food directed at Chinese consumers were for unhealthy food products, in particular fast food restaurants and sweetened beverages. The result of small percentage (11.0%, *n* = 38) for the unhealthy foodstuffs contain nutrients or healthiness reference was not consistent with the previous findings (ex. [38,45,50]). While there is considerable literature exploring the impact of the similar crisis in the West (e.g., [40,51]), the Chinese experience is both delayed and more rapid, and it is taking place under different cultural, economic and political circumstances. 

In contrast to studies considering cereals to be unhealthy (e.g., [35]), this study counted cereals among the healthy foodstuffs since the product contained whole grain cereals by checking ingredients list. The justification also rests on the fact that they usually contained whole grain oat ingredients, were not high in sugar, and were relatively high in fibre. Cereals contribute up to 70% of a person’s energy intake and are highly beneficial to health; however, people in China have very limited choices, and the average cereal intake has decreased over the past 20 years [53,56]. 

The numbers of healthy and unhealthy beverage products advertised on TV were similar (54:59), but a higher proportion (75%) of sugared beverages was provided by local brands (e.g., Huiyuan). All sampled coffee drinks were considered to be unhealthy beverages due to their low nutritional value and, more importantly, their consumption being associated with the addition of sugar, artificial colouring, and flavours enhancer by checking ingredients list. Numerous ads for Chinese specialty beverages promoted the health claims of their products. Unsweetened herbal tea, for instance, promised to have a cooling effect on the body, but proof is scant. Because of the proven connections with the previous literature (e.g., [41,44,50]), our findings lent support to the demand to call attention to insufficient and distorted health-related information on beverage products, which could result in consumer perceptions being misled regarding a food product’s nutritional value.

### 4.2. Marketing Claims with Implicit Messages

In contrast to the American findings, this study showed that the majority of unhealthy food ads in China emphasizing appeals of strength enhancement (67.4%, *n* = 234) were second only to an emphasis on the novelty of the product (85.9%, *n* = 298). The overall Chinese TV ads focused the least on achievements (18.5%, *n* = 89). Another component of implicit messaging that was not readily apparent to the public regarding the overall tactics advertisers employed was the large amount of advertising repetition (64.8%, *n* = 311) and peer popularity (58.1%, *n* = 279). In addition, the most significant difference in advertising appeals occurred in terms of the association between the products and nutrients, which occurred more often with local brands (39.7%, *n* = 79%) than with global brands (9.3%, *n* = 26). Advertising exposure might lead not only to brand awareness but also to increased consumption of energy-dense products, and it has the potential to increase food consumption across various food categories. Food ads could affect food systems and health in ways that are not readily apparent to governments, policymakers, or the public. 

### 4.3. Increased Risk of Obesity

In accordance with the previous study examining TV ads for snack foods affecting the increase in obesity of Chinese children [25,57], food and beverage ads on TV influence people’s dietary habits in several ways. Firstly, the majority of child-targeted food ads took a branding approach. TV advertising plays a crucial role in cultivating the dietary habits of consumers with brand awareness. Boyland and Halford [22] argue that food manufacturers primarily aim to build brand awareness and brand loyalty. It is believed that brand preference precedes purchase behaviours, particularly for children and young people. Most consumers in China are easily attracted by the marketing efforts of globally branded food products. This is especially true for unsophisticated consumers whose perceptions of foreign brands are mainly determined in terms of their symbolic value [27,29]. 

Secondly, the emotional benefits associated with Chinese foodstuffs prevailed, even though the literature highlighted the importance of avoiding food overconsumption as a means of mood management [52]. Several ads portrayed the enjoyment of foods with large package sizes as quick relief from emotional suffering. A local brand, Dabaitu (Shanghai, China), modelled snacking on mouthfuls of white creamy candies to relieve boredom; a fast food giant, McDonald’s, encouraged consumers to eat large portions of burgers and fries to improve their bad mood. Food intake does not necessarily contribute a great amount of fat to the overall diet, but is predictive of a certain type of calorie rich but low-nutrition dietary pattern that increases the risk of obesity.

Last but not least, the tactic of premium offerings which has proven to be effective with consumers [35,58,59] appeared in a large proportion (83.8%) of unhealthy food promotion in China. The food and beverage ads in China were inclined to frequently adopt cartoon characters, mascots, and promises of free gifts and coupons, as rewards for purchasing products. When an advertised product category showed dynamic characters and tie-in tactics, children frequently desire the item over the food. 

This study had several limitations. Firstly, food brands were selected, and ads associated with these brands were then obtained. This may have affected certain outcome variables of interest, as certain global brands may have been in partnership with local brands for manufacturing and cooperative advertising. Indeed, the study was conducted at both a brand level and a product category level, and there may be a complex relationship between the two variables. 

Secondly, exposure to TV advertising does not necessarily equate with being influenced by it. Commercial advertising and food cues may contribute to eating patterns, but the effects of TV messages on behaviour may be far more subtle. Vivid TV advertising images may cause changes in human behaviours, as well as emotions, of which young TV audiences may be unaware. 

Third, these cross-sectional data were retrieved some years ago. A more recent and comparative study could enrich the approach towards studying the risk of obesity in the related stakeholders involved as well as open interrogation that must be resolved systematically. Future studies would do well to include methods that directly address the above points, and it is essential to determine potentially universal factors, as well as factors that are culturally defined, in examining the relationships between ads, food product consumption, and rates of obesity. A longitudinal approach would provide more robust indications concerning the causal links involved. 

## 5. Conclusions

The rapid and extraordinary economic growth in China of the past two decades has also seen changes in eating habits, choices of foods, frequency of eating, and daily total fat and calorie intake, with the result being an alarming increase in prevalence of overweight and obesity. Thus, it is imperative to investigate the advertising strategies of the major food companies in China while taking full advantage of the opportunities created by the country’s rapid economic growth. The challenge involves analysing the factors that drive consumers and present unhealthy products together with health-related messages in the Chinese context. This paper represents a major contribution to our understanding of the obesogenic food context by focusing on advertising appeals and associated factors. Three major themes were discernible in analysing the ads, including the eating context, associations of a food product with increased popularity, and suggestions that a food product might enhance one’s mood.

## Figures and Tables

**Table 1:** Types and frequency of food products advertised associated with brands.

Food Product	No. of Ads	No. of Ads for Local Brands	No. of Ads for Global Brands
	*N* = 480 (%)	*n* = 30 (%)	*n* = 12 (%)
Unhealthy	347 (72.3)	55 (74.3)	19 (25.7)
Fast food	175 (36.5)	0 (0)	3 (100.0)
Sweetened drinks	54 (11.3)	21 (75.0)	7 (25.0)
Cookies/desserts/snacks	50 (10.4)	11 (84.6)	2 (15.4)
Convenient processed food	37 (7.7)	10 (83.3)	2 (16.7)
Candy/chocolate/gum	31 (6.5)	13 (72.2)	5 (27.8)
Healthy	133 (27.7)	19 (65.5)	10 (34.5)
Fruit juice/vegetable juice/water	59 (12.3)	11 (64.7)	6 (35.3)
Dairy	57 (11.9)	4 (80.0)	1 (20.0)
Rice/grain/cereals/pasta	13 (2.7)	2 (66.7)	1 (33.3)
Bread	4 (0.8)	2 (50.0)	2 (50.0)
Total	480 (100)	74 (71.8)	29 (28.2)

**Table 2:** Assessment of advertising tactics and advertising appeals of health-related information categorized as healthy and unhealthy food group in China.

	Healthy Food *n* = 133		Unhealthy Food *n* = 347		Total Ads *N* = 480	
**Advertiser’s Tactics ^1^**	***n***	**%**	***n***	**%**	***N***	**%**
Product demonstration	111	83.5	113	32.6	224	46.7
Ad repetition	98	73.7	240	69.2	338	70.4
Celebrity endorsement	59	44.4	70	20.2	129	26.9
Peer popularity	45	33.8	214	61.7	259	54.0
Premium offerings	33	24.8	289	83.8	322	67.1
Humor employed	28	21.1	248	71.5	276	57.5
Subtotal	374		1174		1548	
**Advertising Appeals ^2^**	***n***	**%**	***n***	**%**	***N***	**%**
Nutrients	87	65.4	38	11.0	125	26.0
Achievement	68	51.1	21	6.1	89	18.5
Taste/flavor/smell/texture	59	44.4	202	58.2	261	54.4
Strength/speed/power	54	40.6	234	67.4	288	60.0
Convenience	26	19.5	199	57.3	225	46.9
Novelty	17	12.8	298	85.9	315	65.6
Subtotal	311		992		1303	

^1^ $x^{2}$ = 186.02, df = 5, *p* < 0.01, ^2^ $x^{2}$ = 361.35, df = 5, *p* < 0.01.

**Table 3:** Assessment of advertising tactics and advertising appeals of health-related information categorized as local and global brand food group in China.

	Local Brand *n* = 199		Global Brand *n* = 281		Total Ads *N* = 480	
**Advertiser’s Tactics ^1^**	***n***	**%**	***n***	**%**	***N***	**%**
Ad repetition	148	74.4	163	58.0	311	64.8
Peer popularity	113	56.8	166	59.1	279	58.1
Celebrity endorsement	80	40.2	49	17.4	129	26.9
Product demonstration	70	35.2	140	49.8	210	43.8
Humor employed	40	20.1	121	43.1	161	33.5
Premium offerings	8	4.0	69	24.6	77	16.0
Subtotal	449		708		1167	
**Advertising Appeals ^2^**	***n***	**%**	***n***	**%**	***N***	**%**
Taste/flavor/smell/texture	110	55.3	157	55.9	267	55.6
Nutrients	79	39.7	26	9.3	105	21.9
Strength/speed/power	32	16.1	31	11.0	63	13.1
Novelty	23	11.6	113	40.2	136	28.3
Achievement	16	8.0	17	6.0	33	6.9
Convenience	4	2.0	26	9.3	30	6.3
Subtotal	264		370		634	

^1^ $x^{2}$ = 81.22, df = 5, *p* < 0.01; ^2^ $x^{2}$ = 95.71, df = 5, *p* < 0.01.

**Table 4:** Assessment of the presentation manner of ads used by the healthy and unhealthy food products in China.

	Healthy Food *n* = 133		Unhealthy Food *n* = 347		Total Ads *N* = 480	
**Audio-Visual Technique ^1^**	***n***	**%**	***n***	**%**	***N***	**%**
Slogan	111	83.5	299	86.2	410	85.4
Character/Mascot	59	44.4	70	20.2	129	26.9
Animation	57	42.9	90	25.9	147	30.6
Jingle	33	24.8	259	74.6	292	60.8
Ties-In	15	11.3	35	10.1	50	10.4
Subtotal	275		753		1028	
**Primary Theme ^2^**	***n***	**%**	***n***	**%**	***N***	**%**
Happiness	89	66.9	259	74.6	348	72.9
Family Togetherness	88	66.2	61	17.6	149	31.0
Peer Togetherness	56	42.1	289	83.3	345	71.9
Mood Alterations	36	27.1	301	86.7	337	70.2
Product performance	28	21.1	245	70.6	273	56.9
Subtotal	297		1155		1452	

^1^ $x^{2}$ = 70.43, df = 4, *p* < 0.01; ^2^ $x^{2}$ = 183.10, df = 4, *p* < 0.01.

## Supplementary Materials

The following are available online at www.mdpi.com/1660-4601/15/1/70/s1. The list of 42 brands included 12 global and 30 local brand’s names in both English and Chinese for further identifying the sampled TV ads. (The sampled 42 brands were available online at www.adzop.com in 2012.)

## Appendix A

**Table A1:** Coding Categories and Inter-coders’ Reliability.

**Brand Origin (Global vs. Local)** (1.0)
Global brand means the advertiser is from the World Brand Lab; while the advertiser of local brand is from China brands database.
**Product Categories** (0.9)
Fast food (restaurants), sweetened drinks (<30% fruit) and soda drinks, fruit juice/vegetable juice/water, other beverages (coffee/tea/energy drinks/soft drinks/others), cookies/desserts/snack foods, convenient processed food (pre-prepared food/processed meals/readymade food/frozen meals for heating up/others), candy/chocolate/gum, dairy (milk/soybean milk/yogurt/others), rice/grain/cereals (cornflakes, muesli)/pasta, meat/fish/poultry, breads, cakes/pastries, condiments, others.
**Health-Related Claims**: Yes/No (0.88)
Yes: if advertisements provided a visual presentation or verbal statement indicating a beneficial relationship between a nutrient or other healthy substance in the product and/or brand. At least one of these criteria needed to be met in order for the advertisement to be coded.
**Healthy vs. Unhealthy food (0.92)**
Healthy food: government recommends this food, comprising essential vitamins and minerals, or nutritional levels of saturated fat, sodium, and sugar.
Unhealthy food: is food that we need to limit our intake of, or consume in moderate proportions to prevent overweight and obesity (high in undesirable nutrients-fat, sugar, salt- and or energy).
**Advertiser’s Tactics**
Ad repetition: is often repeated by broadcasting frequently on TV (0.81)
Product demonstration: show how a branded product works; tangible features only without associated with free gifts or material benefits (0.90)
Peer popularity: product consumption is associated with peer acceptance or being better than one’s peer. It also shows young viewers who are playing with each other (0.91)
Premiums offering: show a freebie when purchasing a product (0.90)
Humor employ: the narrative is supposed to be funny for the viewer (0.93)
Celebrity endorsement: involves a well-known person using his or her fame to help promote a product (0.94)
**Persuasive Appeals**
*Product appeals*
Novelty: new launch, feature of the product is new (0.91)
Nutrients: claim the product is nutritious or provide a visual presentation or verbal statement indicating a beneficial relationship between a nutrient or other substance in the product (0.93)
Convenience: product is accessible, easy to prepare or consume (0.91)
Taste/flavor/smell/texture: description of product’s sensory characteristics (0.92)
*Emotional appeals*
Strength/speed/power: product consumption will enhance physical performance, speed, or energy (0.97) (e.g., sports performance, stamina)
Achievement: product consumption is linked with being better than one’s peers or able to obtain a desired goal (0.88)
Others: Adult approval or disapproval; magic/fantasy; miscellaneous (0.81)
**Presentation Manner**
**Audio-visual techniques** (can code more than one technique)
Animation: use animation elements, both 2-D and 3-D (0.90)
Jingle: the product has to be mentioned, praised, described, or otherwise endorsed in the song (0.97)
Slogan: a catchphrase shown textually or mentioned verbally (0.94)
Tie-ins: a tie-in with a movie, TV show, website or other activities (0.98)
Character/mascot: involving a well-known mascot or cartoon character the average viewer would recognize (0.98)
**Primary Theme**
Happiness: the general mood of the ad is happy; avoids any significant description of the product itself (0.98)
Product performance: reflects emphasis on the product’s particular tangible and intangible features or capabilities (0.92)
Mood alterations: include surprise, create/enhance positive feelings or remove negative feelings (0.91)
Peers togetherness: product consumption is associated with making friends, fitting in, and peer acceptance (0.98)
Family togetherness: product is beneficial to all ages or the consumption is associated with happy family (0.98)
