# Supplementary material for: Implicit Messages Regarding Unhealthy Foodstuffs in Chinese Television Advertisements: Increasing the Risk of Obesity
Source: Int J Environ Res Public Health. 2018 Jan 4;15(1):70. doi: 10.3390/ijerph15010070 (PMC5800169; doi:10.3390/ijerph15010070)
Supplement: Supplementary file 1 [file ijerph-15-00070-s001.pdf]

Supplementary Materials:

The list of 42 brands included 12 global and 30 local brand's names in both English and Chinese for further identifying the sampled TV ads. (The sampled 42 brands were available online at www.adzop.com in 2012.)

12 Global brands

1. Cadbury（吉百利）
2. Coca cola(可口可樂)
3. Danone（達能）
4. Hershey's（好時）
5. Kentucky Fried Chicken (KFC)（肯德基）
6. Kraft（卡夫）
7. Mars（瑪氏）
8. McDonald's（麥當勞）
9. Nestle（雀巢）
10. Pesi（百事）
11. Pizza Hut（必勝客）
12. Wrigley（箭牌）

30 local brands

1. Bright Dairy（光明）
2. Dali（達利）
3. Dabaitu（大白兔）
4. Huiyuan（匯源）
5. Holiland（好利來）
6. Jinmailang（今麥郎）
7. Jinsihou（金絲猴）
8. Laoshan（嶗山）
9. Le Conte（金帝
10. Lolo（露露）
11. Master Kong（康師傅）
12. Nongfu shanquan（農夫山泉）
13. Panpan（盼盼）
14. Qiaqia（恰恰）
15. Sanquan（三全）
16. Sanyuan（三元）
17. Shuanghui（雙匯）
18. Synear(思念）
19. Shengyuan（聖元）
20. Tongyi(統一)
21. Wahaha（娃哈哈）
22. Wanglaoji（王老吉）
23. WantWant（旺旺）
24. Weiwei（維維）
25. Woundersun（完達山）
26. Xizhilang（喜之郎）
27. Xufuji（徐福記）

28. Yaolan（搖籃）
29. Yili (伊利)
30. Yinlu（銀鷺）
